# Epi-illumination gradient light interference microscopy for imaging opaque structures

**DOI:** 10.1038/s41467-019-12634-3

**Published:** 2019-10-16

**Authors:** Mikhail E. Kandel, Chenfei Hu, Ghazal Naseri Kouzehgarani, Eunjung Min, Kathryn Michele Sullivan, Hyunjoon Kong, Jennifer M. Li, Drew N. Robson, Martha U. Gillette, Catherine Best-Popescu, Gabriel Popescu

**Affiliations:** 1 Department of Electrical and Computer Engineering, University of Illinois at Urbana-Champaign, Urbana, IL USA; 2 Beckman Institute, University of Illinois at Urbana-Champaign, Urbana, IL USA; 3 Neuroscience Program, University of Illinois at Urbana-Champaign, Urbana, IL USA; 4 Rowland Institute at Harvard University, Cambridge, Cambridge, MA USA; 5 Department of Bioengineering, University of Illinois at Urbana-Champaign, Urbana, IL USA; 6 Department of Chemical and Biomolecular Engineering, University of Illinois at Urbana-Champaign, Urbana, IL USA; 7 Car R. Woese Institute for Genomic Biology, University of Illinois at Urbana-, Champaign, IL USA; 8 Department of Cell & Developmental Biology, University of Illinois at Urbana-Champaign, Urbana, IL USA

**Keywords:** Optical imaging, Imaging and sensing, Interference microscopy, Phase-contrast microscopy

## Abstract

Multiple scattering and absorption limit the depth at which biological tissues can be imaged with light. In thick unlabeled specimens, multiple scattering randomizes the phase of the field and absorption attenuates light that travels long optical paths. These obstacles limit the performance of transmission imaging. To mitigate these challenges, we developed an epi-illumination gradient light interference microscope (epi-GLIM) as a label-free phase imaging modality applicable to bulk or opaque samples. Epi-GLIM enables studying turbid structures that are hundreds of microns thick and otherwise opaque to transmitted light. We demonstrate this approach with a variety of man-made and biological samples that are incompatible with imaging in a transmission geometry: semiconductors wafers, specimens on opaque and birefringent substrates, cells in microplates, and bulk tissues. We demonstrate that the epi-GLIM data can be used to solve the inverse scattering problem and reconstruct the tomography of single cells and model organisms.

## Abstract (web summary)

Quantitative phase imaging techniques have been limited by multiple scattering of light or its use in transmission mode. Here, the authors show a gradient light interference microscopy method in a reflection geometry which allows for label-free phase imaging of bulk and opaque samples.

## Introduction

Deep tissue optical imaging is fundamentally limited by multiple light scattering. Confocal fluorescence microscopy has become a tool for this type of imaging by employing a focused illumination and a small pinhole in the detection plane, which blocks the out-of-focus light, essentially high-pass filtering the image^1^. Two-photon microscopy achieves deeper penetration due to the larger excitation wavelength, which reduces scattering, as well as a tighter focus resulting from the accompanying nonlinear interaction^2^. More recently, Xu and colleagues showed that tailoring the laser source wavelength and using three-photon excitation extends further the penetration depth for deep-brain tissue imaging^3^. Fluorophore-based imaging yields high specificity with relatively low background noise. However, fluorescence microscopy suffers from limitations as well. The excitation irradiance levels can be very high, especially for nonlinear microscopy, which results in phototoxicity^4,5^. Furthermore, photobleaching limits the duration of continuous imaging that is possible before the fluorophores quench^6^.

As an alternative, photoacoustic imaging combines optical excitation with ultrasound detection to achieve deep penetration depth with high contrast and resolution^7^. High-contrast imaging in thick tissues has been achieved by detecting multiple scattered waves using an oblique illumination^8^. Optical coherence tomography (OCT) is an established label-free imaging technique, which provides depth-sectioning via low-coherence interferometry^9^. Characterized by low phototoxicity and no photobleaching, OCT has opened-up new directions of biomedical investigation and is now a standard clinical procedure in ophthalmology. Full-field OCT provides scattering contrast without the need for raster scanning^10^. Typical OCT records reflectivity from tissue, generating intrinsic contrast in samples that present amplitude modulation. Measuring the phase of OCT signals has enabled solving scattering inverse problems^11^. OCT is the precursor to phase-resolved imaging, which yields high-contrast images even from very transparent structures (see, e.g., Chapter 7 in ref. ^12^). Thus, quantitative phase imaging (QPI)^12^ has experienced tremendous progress, especially over the past decade, enabling numerous biomedical applications^13^. These instruments have been used to monitor time-lapse cell growth^14,15^, studying gametes^16–18^, screening large fields of view^19,20^, imaging blood cells^21,22^, and cancer pathology^23–26^. Although currently employed primarily on weakly scattering specimen^27–33^, recently it has been shown that phase imaging can be extended to multiple scattering specimens^18^. Gradient light interference microscopy (GLIM) combines phase shifting and white light interferometry in a Nomarski geometry to image thick, unlabeled tissues, such as embryos and spheroids^18^. The broadband field illumination in GLIM lead to a strong coherence sectioning effect, causing multiple scattering fields to contribute incoherently to the image. By using phase shifting, the interferometric signal is decoupled from the incoherent, non-modulating background due to multiple scattering, enabling us to reconstruct the scattering potential under the first order Born approximation. While GLIM manages to suppress multiple scattering light and, thus, boost the resulting contrast, it employs a transmission geometry and, as such, is ultimately limited by the thickness of the specimen under investigation. For example, bulk (e.g. in vivo) tissues or specimens placed on opaque substrates cannot be imaged by GLIM.

Here, we present epi-GLIM, a instrument capable of imaging thick unlabeled samples in a reflection geometry. Epi-GLIM shares the capability of multiple scattering suppression with the transmitted light instrument but extends applications to bulk and opaque samples. The instrument is implemented as an add-on module to an existing reflection Nomarski system, and the software developed in-house allows for full automation and scanning in x, y, and z. We demonstrate epi-GLIM’s operation by imaging standard microbeads and nanofabricated structures. The acquisition and mosaicking software developed in-house allows for analyzing very large fields of view, which we illustrated by imaging a 45 cm^2^ portion of a silicon wafer. Exploiting the phase information provided by epi-GLIM, we extracted the nanoscale topography of the wafer across the entire field of view. Furthermore, we demonstrated live cell imaging in microplates and plastic substrates, which are incompatible with transmission imaging. We used epi-GLIM to investigate a morphologically heterogeneous culture of spheroids and extracted the thickness of each quantitatively. The instrument enabled us to image a tendon from an intact mouse leg, which would have been impossible in transmission. Finally, we showed that epi-GLIM can be used to acquire time-resolved tomography of an entire live zebrafish.

## Results

### Epi-GLIM system

**Fig. 1:**
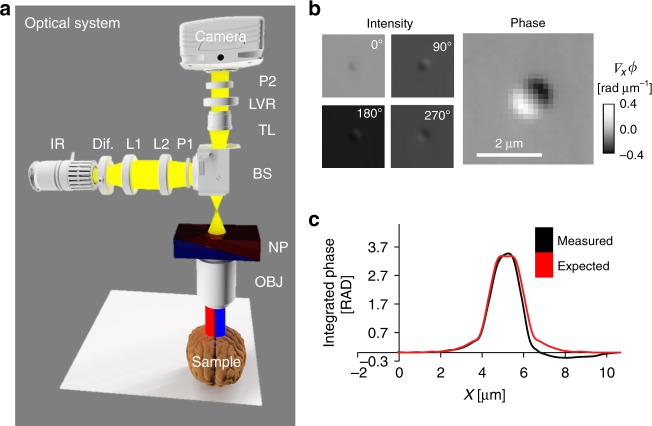
Epi-GLIM enables phase imaging of opaque structures. **a** The epi-GLIM module intercepts the sheared beams in reflected light DIC (blue/red) before they are recombined at the output analyzer (P2). Specifically, we focus light from an IR source through a diffuser (Dif.) by way of lenses L1, L2, beam splitter and objective (L1, L2, BS OBJ). To achieve differential interference contrast, the light is polarized by an input polarizer (P1) and split with a Nomarski prism into two laterally sheared polarizations (NP). The backscattered field is then collected by the objective and tube lens (TL). By using a liquid crystal variable retarder (LVR), we control the relative phase shifts between these polarizations. Rendering performed in Blender. **b** We reconstruct a relief style phase map using a sequence of DIC intensity frames acquired at 0°, 90°, 180°, 270° phase shift, as indicated. **c** To validate our approach, we imaged a 1.9 μm bead on a reflective surface. Upon integration, we recover the expected phase profile for a polystyrene bead blurred by the impulse response of the system (40×/0.75 490 nm), which is twice the value as expected in a transmission geometry. The slight mismatch on the right lobe motivates the use of a measured impulse response in later portions of this work

Epi-GLIM intercepts the sheared beams in DIC to measure the phase gradient across the sample (Fig. 1). In our design, we introduce controlled phase shifts between the two beams ($\varepsilon _n \approx n\frac{\pi }{2}$) using a liquid crystal variable retarder located at the output port of the microscope (for details on calibrating the retarder, see Supplementary Note 1). The intensity measured at the detector becomes^18^

1 $$\begin{array}{l}I_n\left( {\boldsymbol{r}} \right) = I\left( {\boldsymbol{r}} \right) + I\left( {{\boldsymbol{r}} + \delta {\boldsymbol{r}}} \right) + 2\sqrt {I\left( {\boldsymbol{r}} \right)I\left( {{\boldsymbol{r}} + \delta {\boldsymbol{r}}} \right)} {\mathrm{cos}}\\ \left[ {\phi \left( {{\boldsymbol{r}} + \delta {\boldsymbol{r}}} \right) - \phi \left( {\boldsymbol{r}} \right) + \phi _b + \varepsilon _n} \right]\end{array}$$

where ${\mathrm{\Delta }}\phi = \phi \left( {{\boldsymbol{r}} + \delta {\boldsymbol{r}}} \right) - \phi \left( {\boldsymbol{r}} \right) \approx \nabla _x\left( \phi \right)\delta {\boldsymbol{r}}$ is the phase gradient map of interest, *ϕ*_*b*_ (***r***) is a slant-like background due to the DIC optics, *ϕ*_0_ is a constant offset controlled by the Nomarski prism’s position. The offset due to the Nomarski prism position *ϕ*_0_ is removed when the instrument is correctly calibrated, and the background *ϕ*_*b*_ (***r***) is removed by high-pass filtering the phase image^18^.

To recover the phase from the four intensity images, we chose a general form of the standard phase-shifting interferometry equations^34^ that correctly recovers the phase even in cases when modulation is performed in increments that are slightly different from 90°. We note that this pixel wise subtraction removes the non-modulating incoherent background due to multiple scattering. To validate our instrument, we measured microbeads mounted on a reflective substrate embedded in immersion oil (Fig. 1b, c). When imaged on a reflective surface (mirror), the phase shift is twice the value in a transmission configuration^35^. The expected phase shift from the bead is,

2 $$\phi = 2\frac{{\pi d}}{\lambda }\left( {n - n_0} \right),$$

where *λ* (490 nm) is the wavelength of the illumination, *n* is the refractive index of the object (1.605), *n*_0_ (1.518) is the refractive index of the media, and *d* is the known height of the object (1.9 μm). To integrate the measured phase map, we performed a cumulative sum along the direction of the shear. In this integration we used the shear distance value, *δr* = 0.3 μm, measured separately in transmission for this prism. Thus, our instrument can recover quantitatively the phase shift, up to a small difference attributable to focus and the impulse response of the system (Fig. 1c).

### 2D imaging

**Fig. 2:**
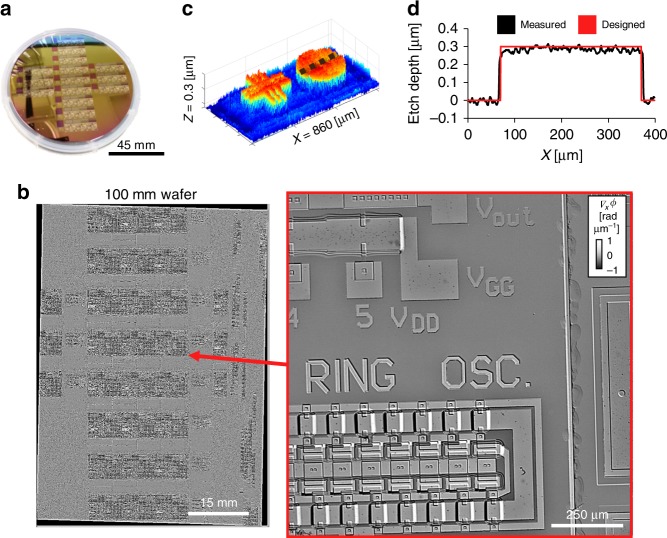
Epi-GLIM enables QPI images of semiconductor wafers. **a** 100 mm semiconductor. **b** 60 × 83 tile mosaic corresponding to a scan range of 45 cm^2^, imaged with a ×10/0.3NA objective. **c** We can measure the system’s impulse response by using a known region of the sample to recover a topographic (integrated) phase map at a different location. **d** After reconstruction, the resulting phase map (large capacitor structures shown) matches well the expected topography

With our epi-GLIM interferometer, we can acquire a large field of view of reflective samples, limited only by the range of the XY translation stage. To demonstrate this capability, we imaged a standard 100 mm diameter semiconductor wafer with a ×10/0.3 objective (Fig. 2) and assembled the resulting mosaic using software developed in-house^36^. In short, this python-based GPU code performs Fourier filtering, sample-free background subtraction, and phase-correlation to determine optical alignment of the mosaic tiles that compose the image. To the best of our knowledge, Fig. 2 is the largest continuous QPI image ever recorded (4563 mm^2^). After this processing, Fig. 2c shows the topography of a capacitor from the wafer compared with the expected (etched) profile.

**Fig. 3:**
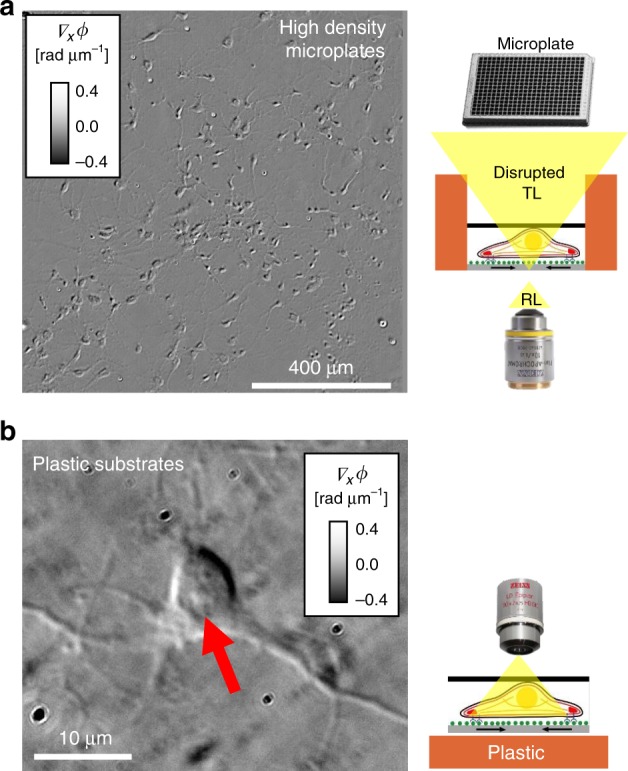
Two challenging samples for transmission DIC **a** Reflected light imaging avoids the meniscus and high walls that make imaging microplates difficult. Representative field of view (×10/0.3) from a 1536 microplate. **b** Birefringent materials such as plastic substrates are incompatible with polarization-based contrast. This problem is overcome in epi-GLIM, by collecting the light before it passes through the birefringent material, revealing intracellular detail such as nucleoli (red arrow). Neurons were imaged after 7 days in vitro (×100/0.75 LD)

The ability to digitize large biological samples is particularly important for plate-reader and phenotypic screening applications. Imaging high-well count plates is difficult with transmitted light modalities as the meniscus and walls surrounding the well often block or distort the illumination (see, for example, ref. ^37^). In Fig. 3a, we show that epi-GLIM can avoid these difficulties by imaging in reflection neurons cultured in a 1536 microplate. Epi-illumination is also useful for applications where the sample is grown on a birefringent material, such as plastic-bottom dishes or 3D printed scaffolds. By illuminating from the top and collecting the backscattered signal, epi-GLIM provides polarization-based contrast even when cells are cultured on normally incompatible substrates (Fig. 3b).

In order to characterize the system’s point spread function, we measured the instrument’s response to a phase step object, i.e., a micropillar obtained by etching quartz. We used a rotating stage to align the micropillar sample orthogonal to the DIC shear direction to maximize contrast. Next, we acquired an axial scan, followed by denoising (see Supplementary Fig. 2). The resulting through-focus series is then rotated and averaged to obtain a measure of the edge response for each focus position. After differentiating the edge response along the lateral direction, we obtain a representation of the PSF at each z-coordinate. A 2D Fourier transform of these data yields the transfer function in the *(k*_*x*_*, k*_*z*_*)* domain.

The advantage of epi-GLIM operating at a fully open condenser compared to the transmission geometry is evidenced by the vast improvement in frequency coverage (Supplementary Fig. 3). As described by the scattering model shown in the Supplementary Note 3, the resulting frequency coverage in epi-GLIM is an autocorrelation of the objective pupil function. Effectively, an open aperture simultaneously captures the frequency coverage that is synthesized in other instruments through multiple measurements^38^. In epi-GLIM, the phase-shifting improves the overall sensitivity to optical pathlength signals^39^. As shown in Supplementary Note 4 and Supplementary Fig. 4 the reflected light geometry leads to a better localized PSF. In practice, the use of the objective as a condenser leads to a higher numeric aperture, giving a further improvement in resolution compared to typical transmitted light instruments.

### 3D imaging

Although the edge spread technique is a sensitive approach to infer the point spread function of the system, each z-stack is only able to collect the PSF along a single plane, i.e., not in 3D. As the shear in GLIM renders the transfer function non-radially symmetric, this approach is informative, but cannot be used to perform 3D reconstructions.

**Fig. 4:**
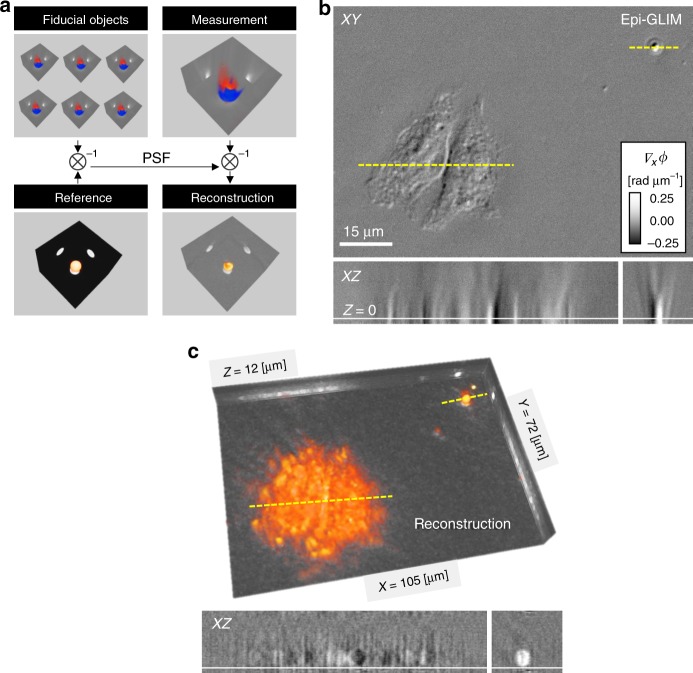
Control object used to reconstruct an unknown sample. **a** The system’s impulse response is measured from a series of known microspheres. When the known shape of the structure is deconvolved from the measured data, the difference contains the system point spread function (PSF). We combined the PSF measurement with the reconstruction step to avoid using two regularizers and recovered the reconstructed volume from subsequent measurements (see Eq. (4)). **b** Epi-GLIM image of a HeLa cell and 3 μm beads before the tomographic reconstruction (×40/0.75). **c** Same field of view after the 3D reconstruction showing the cell and bead with the expected spherical morphology. XZ slices are cut along the yellow dash line

To overcome this challenge, in Fig. 4 we present a 3D image reconstruction technique based on the system’s response to known objects, specifically, microspheres. Essentially, we characterize the system’s point spread function with microspheres measured in the same field of view as the object of interest. This extends the concept of fiducial markers^40^ to tomographic reconstructions. The idea behind our method is that any difference between an object’s known scattering potential *x*_*o*_ (***r***) and the data measured on the microscope *y*_*o*_ (***r***) is due to the system’s PSF, given as:

3 $$y_{o} \left( {\mathbf{r}} \right) =	 {\mathrm{PSF}} \left( {\mathbf{r}} \right) {\circledast} x_{o} \left( {\mathbf{r}} \right) \\ y_{o} \left( {\mathbf{k}} \right) =	 {\mathrm{PSF}}\left( {\mathbf{k}} \right) x_{o} \left( {\mathbf{k}} \right),$$

where ⊛ stands for 3D convolution and the functions of argument **k** are the Fourier transform of those of argument **r**. Therefore, the deconvolution between *y*_*o*_ and *x*_*o*_ yields the PSF. Compared to the sub-diffraction-limited spheres used in fluorescence microscopy, large fiducial objects provide stronger signal and contrast. The proposed technique is applicable to a wide variety of imaging instruments, when the system is linear.

In Fig. 4, we use this fiducial object approach to reconstruct tomograms of adherent cells. We added 3 µm polystyrene microspheres to the standard formalin solution used to fix cells. In this way, the spheres are co-localized with the cells, sharing the same optical path and aberrations^41^. We imaged six spheres from two fields of view and aligned the associated data volumes using the MATLAB Image Registration Toolbox (Fig. 4a). Next, we apply the information from the fiducial object to reconstruct the tomogram of an unknown object (e.g. a cell), of scattering potential *x(****r****)*, from the new data *y(****r****)*. In principle, it is possible to obtain the PSF by comparing the measured data to a high-fidelity simulation of the bead signal and then deconvolve an unknown image. We avoid the need for two regularizers by merging these steps together. Thus, the reconstruction, with the Weiner regularization, in the frequency domain is given by

4 $$\begin{array}{*{20}{c}} {x\left( {\boldsymbol{k}} \right) = y\left( {\boldsymbol{k}} \right)w\left( {\boldsymbol{k}} \right)} \\ {w\left( \boldsymbol{k} \right) = \frac{{y_0^\ast \left( \boldsymbol{k} \right)/x_0^\ast \left( \boldsymbol{k} \right)}}{{\left| {y_0\left( \boldsymbol{k} \right)/x\left( \boldsymbol{k} \right)} \right|^2 + \varepsilon }}} \\ \qquad = 1/{\mathrm{PSF}}\left( {\boldsymbol{k}} \right) \end{array},$$

where *w*(***k***) is the reconstruction filter, *y*(***k***) the measured data in the spatial freqeuncy domain, and *ε* is a regularizer. After this numerical reconstruction, the profile (dashed yellow line) of the sphere co-localized with the cells, displays the correct morphology (spherical shape, Fig. 4c). The overall height of the adhered cell (Fig. 4) is in good agreement with surface plots made of this particular cell line using other imaging systems (atomic force microscopy^42^). We performed an optimization and found that the regularization is optimal for a noise-to-signal ratio of 0.003. This approach generalizes well for all data, such that the same regularization constant was used for the 2D and 3D deconvolution.

**Fig. 5:**
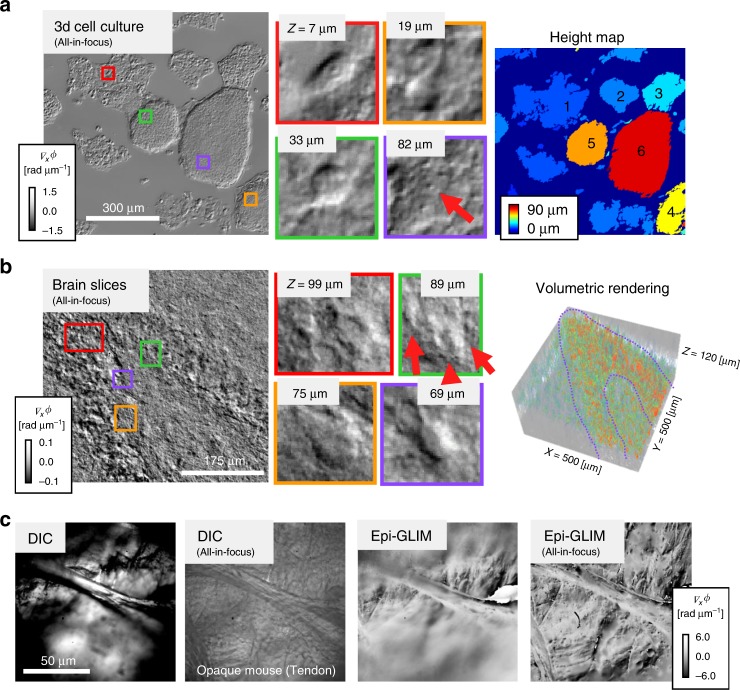
Epi-GLIM for turbid and opaque structures. **a** GLIM reveals cellular level details in a multilayer cell culture (×10/0.3, HepG2 liver cancer spheroids, red arrow nucleoli inside a single cell). By finding the axial section that maximizes the focus, it is possible to recover a height map giving the approximate volume of the spheroids. **b** All-in-focus projection of an acute brain slice imaged in reflection (×20/0.5, ~1.5 μm axial resolution, see Supplementary Fig. 3 for a comparison with theory). As the epi-GLIM reconstruction process suppresses out of focus illumination, small changes to the focus will reveal distinct cellular layers (red arrows pointing to pyramidal neurons). The volumetric rendering highlights the comparably denser crescent of pyramid cells (purple outline). Individual z slices show cellular level detail. **c** Exposed mouse tendon, imaged under a long working distance objective (×100/0.75). Epi-GLIM improves the contrast compared to reflected light DIC at maximum extinction. The difference is accentuated in the all-in-focus image, where the contrast in reflected light DIC is dominated by amplitude contributions which appear as edges in the all-in-focus image

Next, we apply epi-GLIM to larger, more turbid structures that highlight the system’s ability to suppress multiple scattering. This suppression is acomplished by combining phase-shifting with white light interferometry. Specifically, the limited spatial and temporal coherence of the illuminating field rejects multiple scattering by coherence gating. At the same time, the multiple scattering background remains constant during phase shifting and is eliminated upon combining the four intensity images. In Fig. 5a, we investigate a 3D cell culture of HepG2 spheroids, a common model for liver diease^43^. By removing the incoherent background illumination, epi-GLIM shows intracellular detail in these complex cellular systems. To estimate the volume of the spheroids, we acquire a through-focus stack. From this stack, we can reconstruct an all-in-focus image, **Δ***ϕ*, using a commercial software (Helicon Focus), essentially choosing the axial slice that maximizes the detail in a pixel-neighborhood. Thus, we transformed the 3D information from the z-stack into a 2D projection, **Δ***ϕ* (*x, y, z*_max_), where *z*_max_ is obtained by maximizing the variance of the measured **Δ***ϕ* over a window of size *2w*^44^,

5 $$z_{{\mathrm{max}}} = {\mathrm{max}}_{z}\left\{ {\frac{1}{N}}\mathop {\sum }\limits_{i = x - w}^{x + w} \mathop {\sum }\limits_{j = y - w}^{y + w} \Delta \phi ^{2}\left( {i,j,z} \right)- \left[{\frac{1}{N}}\mathop {\sum }\limits_{i = x - w}^{x + w} \mathop {\sum }\limits_{j = y - w}^{y + w} \Delta \phi \left( {i,j,z} \right) \right]^{2} \right\}..$$

In Eq. (5), *N* denotes the number of pixels in the window, and **Δ***ϕ* is the z-stack of epi-GLIM data. This topographic map provides a relatively easy way to obtain the volume of a sample without the need to solve an optical inverse problem. When illuminated under a low numerical aperture, the maximum contrast is typically obtain at the sharp discontinuity between cellular material and surrounding media. In this sample, we note that the largest spheroids are relatively flat, meaning, that their transverse size is larger than their thickness. We found that the thickness difference across different spheroids appears in increments of ~15 µm, suggesting discrete cell layers.

The lack of phase wrapping in this, difficult to image, high-refractive index contrast structure highlights one of the advantages of using the derivative to measure phase shifts. The epi-GLIM data (**Δ***ϕ*) contain the derivative of the phase at each point, which only wraps if there is a steep change in phase across a diffraction spot, i.e. 2pi/(*λ*/2NA). While this derivative phase map, could, in principle, wrap, it is very unlikely for biological specimens and we never observed it. In the case of bulk tissues, coherence gating and phase shifting cause a strong sectioning effect, where the resulting phase image corresponds to a narrow optical slice in the tissue. Thus, each individual z-slice does not suffer from phase wrapping

The ability to see through cellular layers is well suited for working with ex vivo tissues. In Fig. 5b, we show a sliced portion of a rat brain prepared for electrophysiology measurements^45^. Bulk samples are less regularly shaped than samples prepared for transmitted light imaging. Due to epi-GLIM’s high sectioning capabilities, even with low power objectives (×20/0.5), we can recover cellular detail at different layers, such as the neuron shown in Fig. 5b. The 3D rendering of the integrated phase highlights the crescent of granular neurons. Although acute brain slices are typically studied in a transmission geometry, our ability to recover an interferometric signal in a reflection geometry hints at the potential for in vivo applications.

In addition to improving contrast in highly scattering samples, epi-GLIM enables quantitative phase imaging of large biological samples that are opaque. In Fig. 5c, we dissected a freshly euthanized mouse to expose the tendon at the end of the foot. The exposed portion of the tendon was immediately imaged under a long working distance objective (×100/0.75). Due to the sample’s cylindrical profile (irregular profiles are common in bulk tissue), only a narrow portion of the sample appears in focus at each individual plane. Using the same technique as before, we can obtain an all-in-focus projection of the structure by performing an axial scan. Compared to regular DIC imaging, the epi-GLIM image reveals much higher contrast and high-resolution features.

**Fig. 6:**
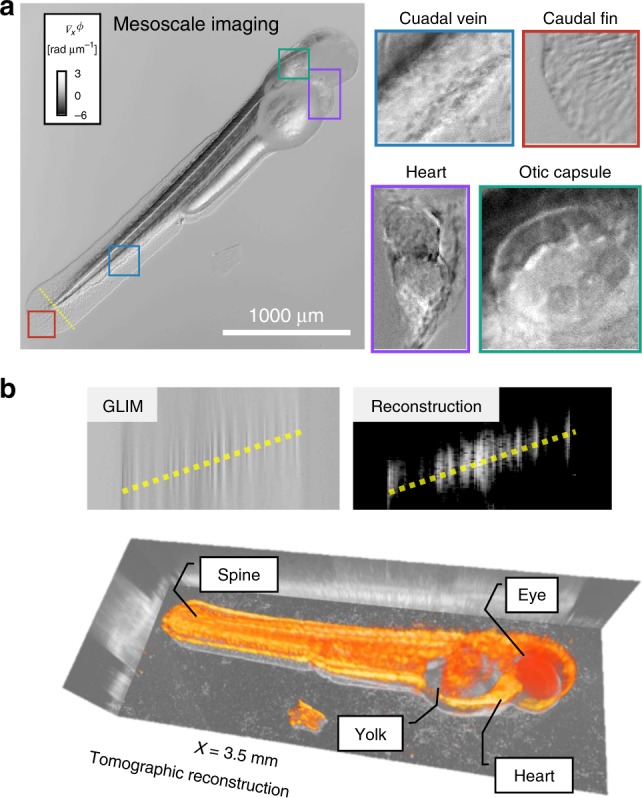
Epi-GLIM for quantitative phase imaging of whole animals. **a** QPI tomogram of a free-swimming larval zebrafish 6 days post fertilization (×5/0.13) with highlighted organ scale structures. **b** Tomographic reconstruction was performed using slice-by-slice high-pass filtering. Due to absorption, the eye reveals a random phase signal, which we replaced with a constant value, for visualization purposes

In addition to inspecting cellular detail in turbid tissue, epi-GLIM is well suited to image entire model organisms at the mesoscopic scale. We demonstrated this capability by imaging a free-swimming zebrafish larva (Fig. 6a). Despite the low numeric aperture of our objective (×5/0.13), we recovered sample features, such as a beating heart, single cells flowing through blood veins (Supplementary Movie 3), cells on the fin, and the ottic capsule. We note that portions of the eyeball were difficult to image due to the high absorption of light. In order to reconstruct the entire organism we used the method outlined in^18^ where high-frequency data is bilaterally filtered. After this reconstruction, it becomes apparent that the animal is resting at a tilt (Fig. 6b, dashed yellow line). Importantly, because we used a full-field imaging system we were able to acquire the whole tomographic series, consisting of 469 slices, before the animal swam away.

## Discussion

In this paper, we demonstrated a epi-illumination phase imaging modality that combines DIC with phase shifting interferometry to suppress multiple scattering. Epi-GLIM shares with OCT^10^ and the oblique back-illumination microscopy^8^ the goals of imaging bulk tissues. Compared to OCT, epi-GLIM uses a common path interferometric geometry, which grants high phase sensitivity. The phase shifting approach allows epi-GLIM to suppress multiple scattering and image with coherent fields, while the oblique illumination method uses multiply scattered light for imaging. In our case, the phase information can be quantified and used to solve scattering inverse problems, as shown in Fig. 4.

Our compact module is well integrated with conventional microscopes enabling us to image large volumes. We demonstrated this capability by retrieving nanoscale topography of a silicon wafer across a 45 cm^2^ field of view. Further, by improving contrast through phase-shifting and using epi-illumination, our approach facilitates imaging of biological structures that would be difficult to perform in transmission. Epi-GLIM can be used with unconventional substrates by imaging cells in a high-count multi-well and on birefringent substrates.

With the incoherent background removed, GLIM enables imaging of turbid structures, such as 3D cellular cultures and free-swimming zebrafish. We also demonstrated that this approach is applicable to opaque samples such as exposed bulk tissue. Epi-GLIM and the reflected light geometry can be used to perform routine inspection tasks on living tissue in an electrophysiology setting. The optimization of our system for in vivo imaging is the subject of current work. Finally, we expect that the fiducial object approach to tomographic reconstruction can be readily extended to other microscopy modalities.

## Methods

### Epi-GLIM module

Epi-GLIM is implemented as an add-on module to a commercial differential interference contrast (DIC) system. In our implementation, the light path begins from an LED source at 780 nm (Fig. 1a, IR). The light is directed toward a diffuser internal to the microscope, facilitating spatially incoherent illumination to completely fill the objective aperture (Fig. 1a, Dif.). This light is polarized by P1 before being directed towards the sample via a beam splitter (Fig. 1a, BS). Next, a Nomarski prism splits the illumination into two fields of orthogonal polarizations (Fig. 1a, NP, red and blue), which are focused onto the sample by the microscope’s objective. These two waves arrive at the sample plane slightly offset (sheared) along one direction, with a spatial shift that is smaller than the diffraction spot (2D point spread function, PSF) of the microscope. Upon backscattering off the sample, the fields are recombined by the Nomarski prism and the image is relayed at the camera through the tube lens (Fig. 1a, TL). Immediately before the camera, the final field is analyzed by a polarizer parallel to the input (Fig. 1a, P2). The resulting image resembles the derivative of the phase map along the shear direction. The Nomarski prism was translated to introduce a phase bias of π, such that the sample signal is measured against a dark background.

To quantitatively measure the phase gradient at each pixel, we augment this design with a variable retarder that modulates the relative offset between the two sheared beams (Fig. 1a, LVR). This modulator is mounted at a 45° with respect to the input polarization. The LVR calibration procedure is shown in Supplementary Fig. 1. For each epi-GLIM image, we acquire four intensity images corresponding to four phase shifts, typically, multiple of π/2, as in typical phase shifting interferometry (Fig. 1b). However, as shown in Supplementary Note 1, our calibration makes the system robust to small variations around these phase shift values. We acquired data at a final throughput of four epi-GLIM images per second, resulting from sixteen intensity frames per second. The rate limiting factor is LVR stabalization time, while all processing, including denoising, is performed in real-time (see Supplementary Note 2).

Reflected light imaging (Figs. 1–6) was conducted on an Axio Imager D2 (Zeiss) upright research microscope with an automated stage and piezo focus. The transmitted light image in Fig. 3 was acquired with a closed condenser on an Axio Observer Z1 (Zeiss).

### Sample preparation and microscopy

To demonstrate the broad applicability of our technique, we investigated a wide range of samples. The wafer shown in Fig. 2 contains a representative sampling of silicon devices. The transparent micro-fabricated quartz pillar used for point spread function measurements in Supplementary Fig. 3 was prepared according to the protocol in^46^.

Primary neurons (Fig. 3) were grown on Poly-d-Lysine (Advanced BioMatrix 5049-50) treated glass and plastic dishes. Dissected cortical tissues from AGE rats were dissociated in 3 mg/mL protease 23 (Sigma P4032) in 1X SLDS (pH 7.4). After a 4-h plating period, the cells were grown in a maintenance media for 7 days. Neurons were fixed with 4% formaldehyde prior to imaging. To image on plastic substrates, the dry, long working distance, objective (×100/0.75) was immersed directly into the media.

The HeLa cells in Fig. 4 were grown according to the protocol in^47^ and fixed with formaldehyde before imaging. The sample was cultured on a 20 mm glass slide placed in a larger 35 mm petri dish (CellVis, D35-20-0-TOP). As discussed in results, we used microspheres as known objects to retrieve the point spread function by a deconvolution technique. Immediately before imaging, a dilution of 3 µm polystyrene beads (Polybead®, 17134-15) was poured over the sample. A coverslip was placed over the petri dish with the cells, and excess liquid was removed from the edges. This creates a tight seal that brings the cell-coated surface of the petri dish into the working distance of the dry objective (40/0.75). Finally, the edges of the coverslip “sandwich” were sealed to avoid evaporation during imaging. Due to the poly-d-lysine coating, the microspheres in (Fig. 4) were stable for the duration of the imaging.

The HepG2 liver cancer cell clusters in (Fig. 5a) were grown according to the protocol in, with the omission of the agar plating step. The sample was fixed in 4% formaldehyde, sealed with nail polish and inverted for imaging.

The acute brain slice in Fig. 5b was prepared from a 4-week-old rat. Following a standard protocol^45^, a coronal hippocampal brain slice was cut to 150-µm on a vibrating tissue slicer and placed into a chamber containing artificial cerebrospinal fluid (ACSF, 95% O_2_/5% CO_2_). The slice was imaged an hour after preparation. To avoid surface reflections, the objective was immersed directly in the ACSF media.

The tendon in Fig. 5c was harvested from a euthanized mouse according to a standard protocol^48^. The animal was placed on a microscope stage, and the exposed tendon was imaged immediately after dissection.

The larval zebrafish in Fig. 6 and Supplementary Movie 3 were prepared according to the protocol in ref. ^49^. The sample was imaged at ~5–6days post fertilization. The animal was kept in the field of view during imaging using the motorized microscope stage.

### Reporting summary

Further information on research design is available in the Nature Research Reporting Summary linked to this article.

## Supplementary information

Supplementary Information

Description of Additional Supplementary Files

Supplementary Movie 1

Supplementary Movie 3

Supplementary Movie 2

Reporting Summary

## Data Availability

The data that support the findings of this study are available from the corresponding author upon reasonable request.

## Code availability

The code and computer algorithms that support the findings of this study are available from the corresponding author upon reasonable request.
