# Supplementary material for: Epi-illumination gradient light interference microscopy for imaging opaque structures
Source: Nat Commun. 2019 Oct 16;10:4691. doi: 10.1038/s41467-019-12634-3 (PMC6795907; doi:10.1038/s41467-019-12634-3)
Supplement: Supplementary file 1 — Supplementary Information [file 41467_2019_12634_MOESM1_ESM.pdf]

Supplementary information for

Epi-illumination gradient light interference microscopy for imaging opaque structures

Supplementary Note 1: LVR Calibration

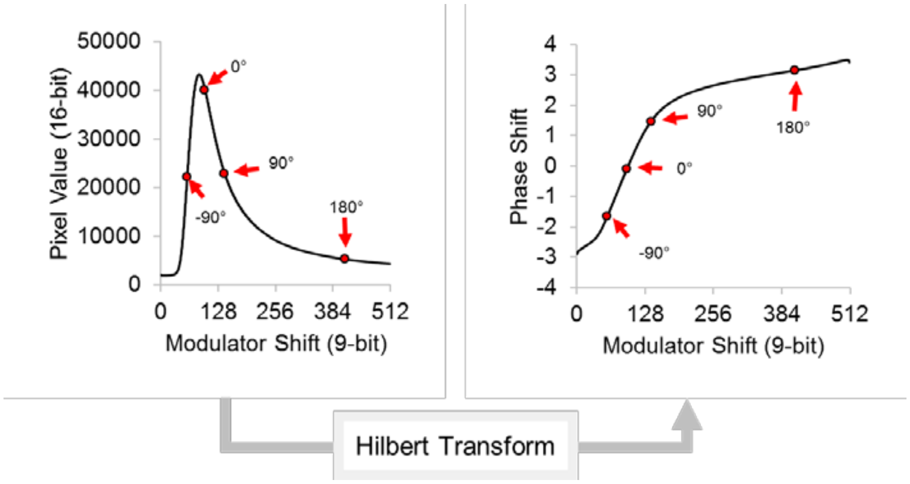

Supplementary Figure 1. Liquid crystal variable retarder calibration procedure. We determine the relationship between the LCVR voltage and phase shift by acquiring all series of images with increasing modulator voltage. The Hilbert transform of this sequence yields the phase shift imparted by the modulator. Red arrows indicate frames at 90° phase shifts used for the four-frame shifting interferometry. The curve was acquired over a [0, 7] volt range using blue (490 nm) illumination.

We determine the relationship between the switching voltage of the variable retarder and the achieved phase shift by performing a calibration on an empty area of the sample. In principle this calibration must be done whenever the spectrum is expected to change, typically for each light source. In this work acquire a sequence of images at increasing modulator voltages, producing 512 samples from 0 to 7 volts. By taking the Hilbert transform of this sequence we can recover the instantaneous phase associated with the gray level on the modulator. Although in principle, three such points can be used for phase-retrieval, for improved signal-to-noise, we use four such points. As discussed in results, in our implementation we derive a precise equation that allows us to use phase-shifts other than 90°. We note that adjustments to the de Sénarmont prism, after calibration, when they do not cause phase-wrapping, appear as a uniform phase offset that is typically removed during background subtraction.

Supplementary Note 2: Real-Time Processing

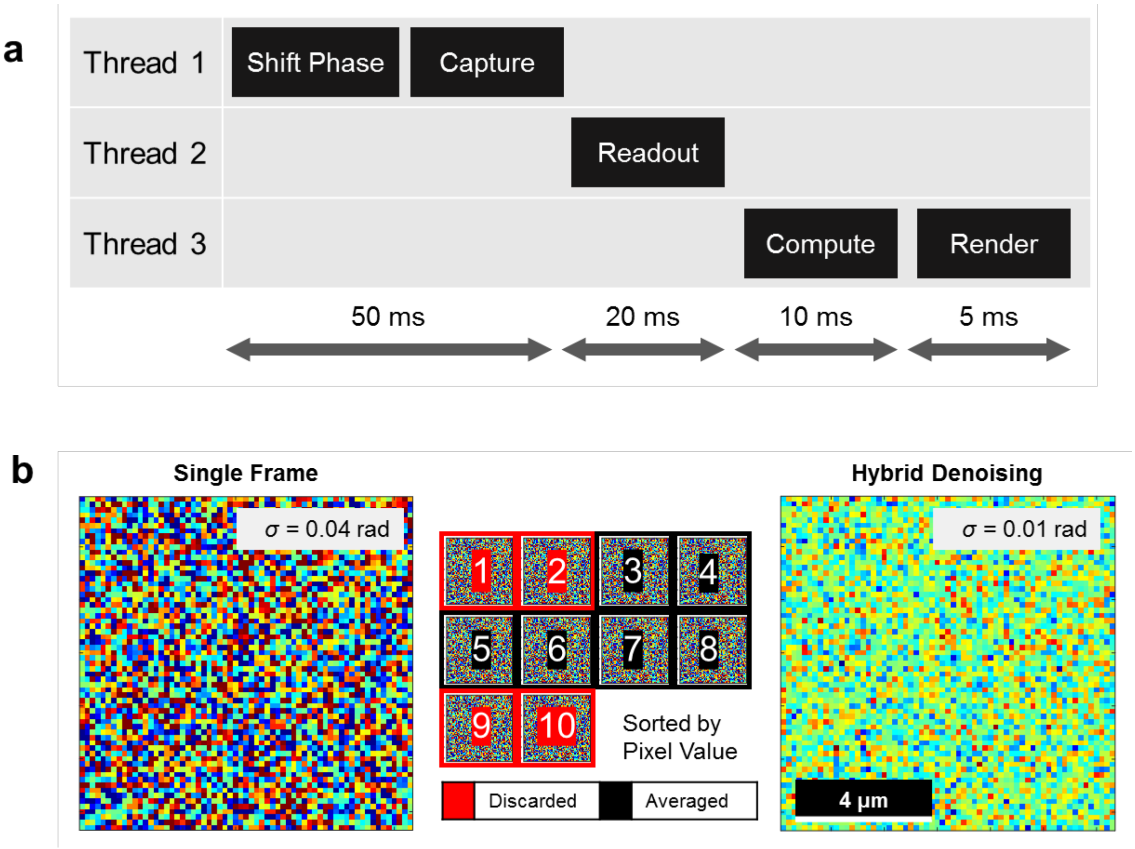

Supplementary Figure 2. Real-time processing and denoising a, Real-time GLIM image processing overlaps computation with image acquisition, rate limited by LVR modulator stability (~40 ms in this work). **b,** To reduce temporally varying noise, the metrology measurements were denoised using a hybrid scheme that combines mean and median filtering where 10 images are combined into a single image. After application of our technique on a sample free image (microscope cover glass) the standard deviation of phase signal (assumed to be noise) is reduced by a factor of four.

Epi-GLIM phase maps are acquired using an in-house image acquisition platform. In our multithreaded implementation, we overlap the phase-shifting process (Supplementary Figure 2, “Thread 1”), with the camera readout, (“Thread 2”) along with the computation and rendering (“Thread 3”). In this way, acquisition is limited by the hardware (phase-shifting and camera exposure times) rather than software. To reduce pixel level noise in the PSF measurements, we multiple phase maps are combined into a single image. to reduce the noise by a factor of four on a

sample free area (40x/0.75). We refer to our technique as “hybrid” denoising as it combines mean and median filtering. Specifically, we acquire 10 phase maps, sort the pixel values using a fast sorting-network, discard the two largest and smallest frames, and average the remaining values. This processing is performed online so as to reduce hard drive requirements. On an empty sample (microscope cover glass), this reduces the standard deviation of the phase by a factor of four. As our hybrid technique was sufficient for our applications, we did not investigate other strategies for noise reduction.

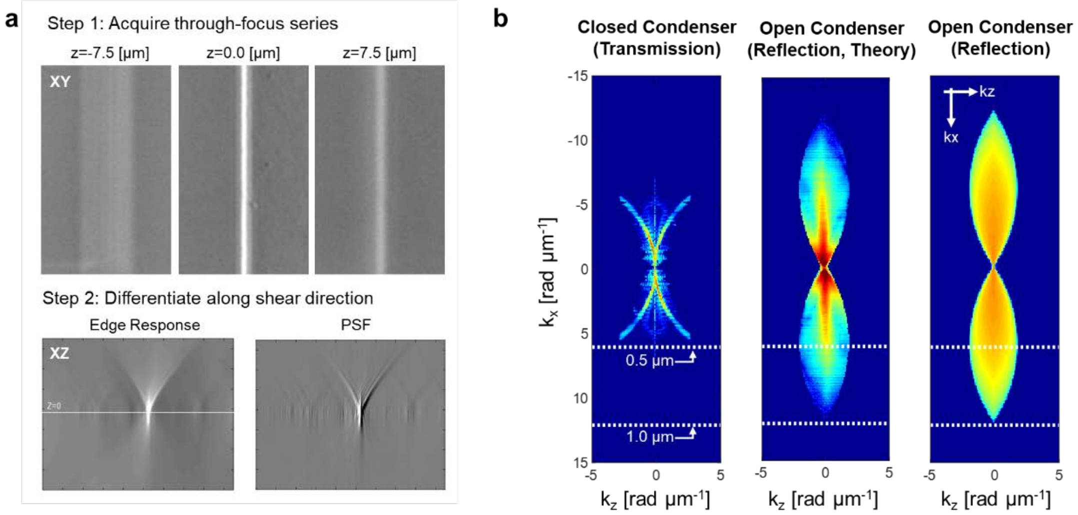

Supplementary Figure 3. Maximizing resolution by spatiotemporally incoherent fields a, The 3D PSF can be estimated using a phase edge. To characterize the system’s impulse response, we acquired a series of through-focus images of a large quartz micropillar (“step 1”). The pillar was aligned to be orthogonal to the DIC shear to maximize the contrast. We collected an edge spread function from each z-slice (“step 2”), which after differentiation along the axial direction gives a point spread function perpendicular to the edge. **b,** The frequency coverage in epi-GLIM is substantially larger than the coverage obtained in transmitted light GLIM with a closed condenser (NAc=0.09). The results agree well with the theory derived in note #3 of Supplementary Information, recovering the resolution limits for coherent and incoherent imaging (0.5 µm vs 1.0 µm, 40x/0.75 at 780 nm). Optical transfer functions are shown on a log10 plot. Frequency bins outside the theoretical coverage given in the supplemental were set to zero. The theoretical plot was normalized to match the power in the measured data.

Supplementary Note 3: Scattering wave propagation in inhomogeneous media

The scattering wave, U_s, in an inhomogeneous media, satisfies the inhomogeneous Helmholtz equation [1, 2]

$$\nabla^2 U_s(\mathbf{r},\omega) + n_0^2\beta_0^2(\omega)U_s(\mathbf{r},\omega) = -\beta_0^2(\omega)\chi(\mathbf{r},\omega)U_i(\mathbf{r},\omega) \tag{1}$$

where **r** is the spatial coordinates, n_0 the refractive index of the background, $\beta_0=\omega/c$, c the speed of light in vacuum, χ the scattering potential of the object, and U_i the illumination. Taking the Fourier transform with respect to **r**, the equation above can be brought into the **k** domain.

$$-k^2 U_s(\mathbf{k},\omega) + \beta^2(\omega)U_s(\mathbf{k},\omega) = -\beta_0^2(\omega)A_0(\mathbf{k}_\perp)\left[\chi(\mathbf{k},\omega)ⓥ_k U_i(\mathbf{k},\omega)\right] \tag{2}$$

In Eq. S2, $\beta= n_0\beta_0$, $ⓥ_k$ represents convolution in the **k** domain, and $\mathbf{k}_\perp$ the projection in the transverse plane. Here we use the same symbol but different arguments for a function and its Fourier transform, e.g., $U_s(\mathbf{k},\omega)$ is the Fourier transform with respect to **r** of $U_s(\mathbf{r},\omega)$. In a microscopic system, A_o is the entrance pupil of the objective, defined as

$$A_o(\mathbf{k}_\perp) = \begin{cases} 1, & |\mathbf{k}_\perp| \le \beta_0\mathrm{NA} \\ 0, & \text{else} \end{cases}, \tag{3}$$

where NA is the numeric aperture. Let U_i be a single plane wave originating at the condenser aperture,

$$U_i(\mathbf{r}_\perp, z, \omega) = A_c(\mathbf{k}_{i\perp},\omega)e^{i\mathbf{k}_{\mathbf{i}\perp}\cdot\mathbf{r}_\perp}e^{i\gamma_i z}, \tag{4}$$

where A_c is the condenser aperture, $\mathbf{r} = (\mathbf{r}_\perp, z)$ is the spatial coordinate, with $\mathbf{r}_\perp$ the transverse coordinate, $\mathbf{k}_{\mathbf{i}\perp}$ the incident light transverse wavevector, conjugate variable to $\mathbf{r}_\perp$, and $\gamma_i = \sqrt{\beta^2 - k_{i\perp}^2}$ is the incident light axial. Following a similar procedure to the one in Ref. [2], the forward and backscattering fields can be easily derived as

$$
\begin{aligned}
U_f\left(\mathbf{k}_\perp, z, \omega\right) &= \frac{\beta_0^2\left(\omega\right)}{2\gamma}\left[A_o\left(\mathbf{k}_\perp\right) A_c\left(\mathbf{k}_{i\perp}, \omega\right) \chi\left(\mathbf{k}_\perp - k_{i\perp}, \gamma - \gamma_i\right)\right] e^{i\gamma z} \\
U_b\left(\mathbf{k}_\perp, z, \omega\right) &= -\frac{\beta_0^2\left(\omega\right)}{2\gamma}\left[A_o\left(\mathbf{k}_\perp\right) A_c\left(\mathbf{k}_{i\perp}, \omega\right) \chi\left(\mathbf{k}_\perp - k_{i\perp}, -\gamma - \gamma_i\right)\right] e^{-i\gamma z}
\end{aligned} \tag{5}
$$

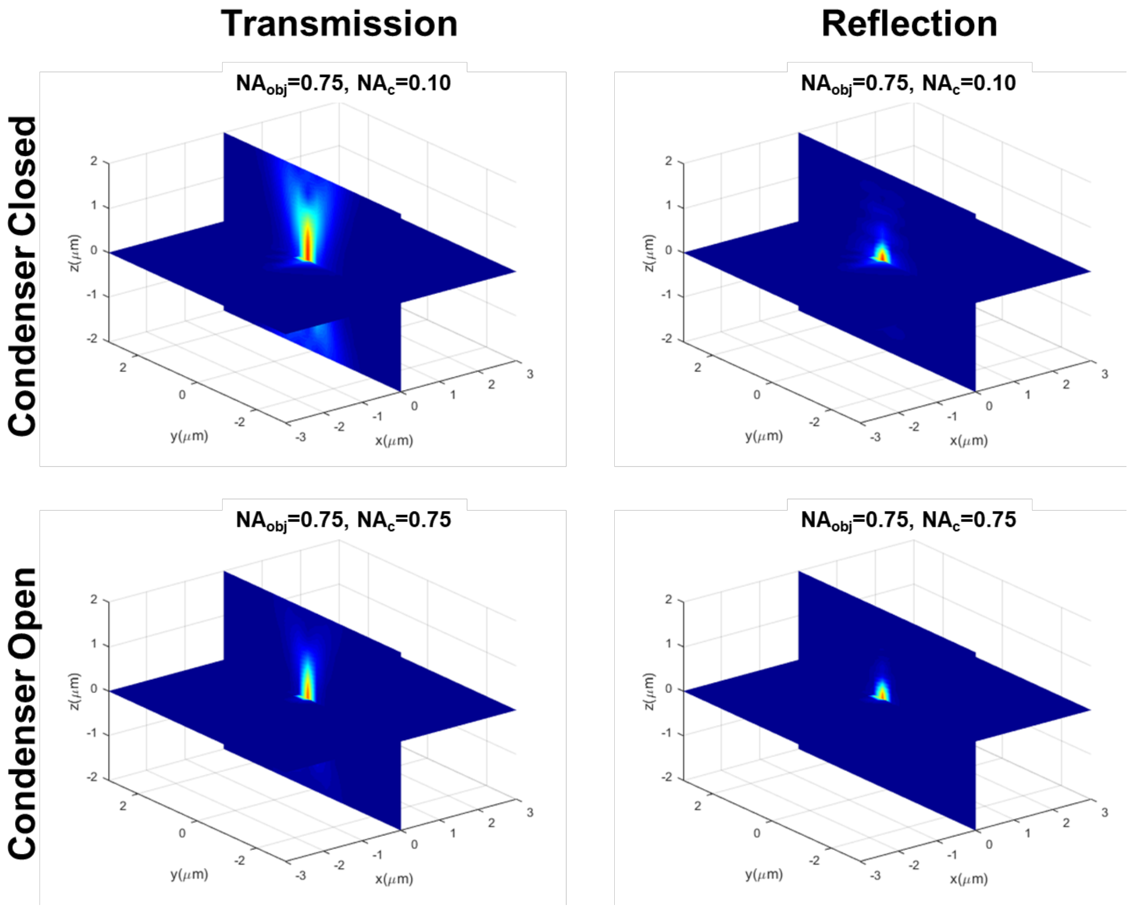

Supplementary Figure 4. PSF is better localized in a backscattering geometry. Representative simulations of a transmission and reflected light system (40x/0.75) with condenser closed (NA=0.10) and open (NA=0.75) showing the mutual intensity. The simulation models broadband illumination uniformly disturbed from 400 nm to 700 nm. In a reflected light geometry (Fig. 1), the illumination diaphragm controls the numeric aperture of the indecent field, effectively serving the role of the tunable pinholes found on transmitted light condensers.

Supplementary Note 4: System transfer function in epi-GLIM

In the differential interference contrast geometry, the intensity image detected by the camera, *I,* is an interference between a total field, U_t, and its copy with a small lateral shift,

$$\begin{aligned} I(\mathbf{r}_\perp, z) &= \left\langle \left| U_t(\mathbf{r}_\perp, z) + U_t(\mathbf{r}_\perp + \Delta x, z) \right|^2 \right\rangle \\ &= \left\langle \left| U_t(\mathbf{r}_\perp, z) \right|^2 \right\rangle + \left\langle \left| U_t(\mathbf{r}_\perp + \Delta x, z) \right|^2 \right\rangle + 2\,\mathrm{Re}\left[\left\langle U_t(\mathbf{r}_\perp, z) U_t^*(\mathbf{r}_\perp + \Delta x, z) \right\rangle \right] \end{aligned} \tag{6}$$

where $\langle \ \rangle$ represents the ensemble average, * the complex conjugate, and Δx indicates the lateral shift, assumed in the *x* direction. In Eq. S6, U_t is the total field, *i.e.* the sum of incident and the scattered field, and thus, the interference term can be expanded as

$$\begin{aligned} \Gamma(\mathbf{r}_\perp, z) &= \left\langle U_t(\mathbf{r}_\perp, z) U_t^*(\mathbf{r}_\perp + \Delta x, z) \right\rangle \\ &= \left\langle U_i(\mathbf{r}_\perp, z) U_i^*(\mathbf{r}_\perp + \Delta x, z) \right\rangle + \left\langle U_s(\mathbf{r}_\perp, z) U_s^*(\mathbf{r}_\perp + \Delta x, z) \right\rangle \\ &\quad + \left\langle U_s(\mathbf{r}_\perp, z) U_i^*(\mathbf{r}_\perp + \Delta x, z) \right\rangle + \left\langle U_i(\mathbf{r}_\perp, z) U_s^*(\mathbf{r}_\perp + \Delta x, z) \right\rangle \end{aligned} \tag{7}$$

We note that light that scatters multiple times will traverse a longer path causing it to exit the coherence gate. In GLIM we aim to achieve the tightest coherence gate through the use of fully open condenser and broadband illumination creating a narrow slice where the optical phenomenon is well modeled by the first-order Born approximation. Thus, under the assumption of the first-order Born approximation, U_i is the dominant field which makes the $\left\langle U_s(\mathbf{r}_\perp, z) U_s^*(\mathbf{r}_\perp + \Delta x, z) \right\rangle$ term in Eq. S7 negligible, and therefore, the information of the object is only contained in the mutual-intensity between the incident and the scattered field, written in the **k**-domain as

$$\begin{aligned} W(\mathbf{k}_\perp, z) &= \Im\left[\left\langle U_s(\mathbf{r}) U_i^*(\mathbf{r}) \right\rangle \right] \\ &= U_s(\mathbf{k}_\perp, z) \circledcirc_{k_\perp} U_i^*(-\mathbf{k}_\perp, z) \end{aligned} \tag{8}$$

In the transmission DIC geometry, U_i is the un-scattered incident light. In backscattering measurement, U_i is the specularly reflected incident light. Using the expression in Eqs. S4-5, at the conjugate plane of the specimen plane, the cross correlation for both transmission and reflection system can be calculated as

$$\begin{aligned} W(\mathbf{k}_\perp, z, \omega)\big|_f &= \frac{\beta_0^2(\omega)}{2\gamma^{'}} A_c^2(\mathbf{k}_{i\perp}, \omega) A_o(\mathbf{k}_\perp + \mathbf{k}_{i\perp}) \chi(\mathbf{k}_\perp, \gamma^{'} - \gamma_i) e^{i(\gamma^{'} - \gamma_i)z} \\ &= \frac{\beta_0^2(\omega)}{2\gamma^{'}} A_c^2(\mathbf{k}_{i\perp}, \omega) A_o(\mathbf{k}_\perp + \mathbf{k}_{i\perp}) \chi(\mathbf{k}_\perp, z) ⓥ_z e^{i(\gamma^{'} - \gamma_i)z} \\ W(\mathbf{k}_\perp, z, \omega)\big|_b &= \frac{\beta_0^2(\omega)}{2\gamma^{'}} A_c^2(\mathbf{k}_{i\perp}, \omega) A_o(\mathbf{k}_\perp + \mathbf{k}_{i\perp}) \chi(\mathbf{k}_\perp, z) ⓥ_z e^{-i(\gamma^{'} + \gamma_i)z} \end{aligned} \tag{9}$$

where $\gamma^{'}$ is the version of γ where $\mathbf{k}_\perp$ is shifted by $\mathbf{k}_{i\perp}$, meaning $\gamma^{'} = \sqrt{\beta^2(\omega) - (k_\perp + k_{i\perp})^2}$. In Eqs. S9, we use the properties of Fourier transforms, where the convolution of a function with a complex exponential results in the Fourier transform of that function multiplied by the complex exponential, namely, $f(z) ⓥ_z e^{iqz} = e^{iqz} f(q)$, and the result is also applied on the backscattering situation, which is not repeated. Notice that the axial frequency coverage in the reflection geometry is larger than in transmission, which indicates that epi-GLIM provides stronger sectioning than GLIM. For a microscopic system, we usually use a system transfer function (or an impulse response) to characterize the system performance, and, therefore, we perform a Fourier transform on Eqs. S9 with respect to z, which yield

$$\begin{aligned} W(\mathbf{k}_\perp, k_z, \omega)\big|_f &= \chi(\mathbf{k}_\perp, k_z) \frac{\beta_0^2(\omega)}{2\gamma^{'}} A_c^2(\mathbf{k}_{i\perp}, \omega) A_o(\mathbf{k}_\perp + \mathbf{k}_{i\perp}) \delta\left[k_z - (\gamma^{'} - \gamma_i)\right] \\ W(\mathbf{k}_\perp, k_z, \omega)\big|_b &= \chi(\mathbf{k}_\perp, k_z) \frac{\beta_0^2(\omega)}{2\gamma^{'}} A_c^2(\mathbf{k}_{i\perp}, \omega) A_o(\mathbf{k}_\perp + \mathbf{k}_{i\perp}) \delta\left[k_z + (\gamma^{'} + \gamma_i)\right] \end{aligned} \tag{S10}$$

Furthermore, since the sample is illuminated by a broadband light source from a variety of angels, the results in Eq. S10 need to be integrated as

$$
\begin{aligned}
W\left(k_{\perp},k_z\right)\Big|_f &= \chi\left(\mathbf{k}_{\perp},k_z\right)\iiint \frac{\beta_0^2\left(\omega\right)}{2\gamma^{'}} A_c^2\left(\mathbf{k}_{i\perp},\omega\right) A_o\left(\mathbf{k}_{\perp}+\mathbf{k}_{i\perp}\right)\delta\left[k_z-\left(\gamma^{'}-\gamma_i\right)\right] d\mathbf{k}_{i\perp} d\beta \\
&= \chi\left(\mathbf{k}_{\perp},k_z\right)\frac{c}{2n_0^3}\int\left[\frac{\beta^2}{\gamma} A_o\left(\mathbf{k}_{\perp}\right)\delta\left(k_z-\gamma\right)\right] ⓥ_k \left[A_c^2\left(\mathbf{k}_{\perp}\right)\delta\left(k_z+\gamma\right)e^{-i\gamma z}\right] d\beta \\
W\left(k_{\perp},k_z\right)\Big|_b &= \chi\left(\mathbf{k}_{\perp},k_z\right)\iint \frac{\beta_0^2\left(\omega\right)}{2\gamma^{'}} A_c^2\left(\mathbf{k}_{i\perp},\omega\right) A_o\left(\mathbf{k}_{\perp}+\mathbf{k}_{i\perp}\right)\delta\left[k_z+\left(\gamma^{'}+\gamma_i\right)\right] d\mathbf{k}_{i\perp} d\beta \\
&= \chi\left(\mathbf{k}_{\perp},k_z\right)\frac{c}{2n_0^3}\int\left[\frac{\beta^2}{\gamma} A_o\left(\mathbf{k}_{\perp}\right)\delta\left(k_z+\gamma\right)\right] ⓥ_k \left[A_c^2\left(\mathbf{k}_{\perp}\right)\delta\left(k_z+\gamma\right)\right] d\beta
\end{aligned}
\tag{S11}
$$

Note that in epi-GLIM, $A_o=A_c$, meaning that the objective acts as a condenser. As shown in Supplementary Figure 4, the resulting $W\left(x,y,z\right)$ is better localized in the axial domain indicating a theoretical improvement in sectioning.

Supplementary References

1. Born M, Wolf E. *Principles of optics : electromagnetic theory of propagation, interference and diffraction of light*, 7th expanded edn. Cambridge University Press (1999).

2. Hu C, Popescu G. Physical significance of backscattering phase measurements. *Opt Lett* **42**, 4643-4646 (2017).
