# Supplementary material for: Epi-illumination gradient light interference microscopy for imaging opaque structures
Source: Nat Commun. 2019 Oct 16;10:4691. doi: 10.1038/s41467-019-12634-3 (PMC6795907; doi:10.1038/s41467-019-12634-3)
Supplement: Supplementary file 2 — Description of Additional Supplementary Files [file 41467_2019_12634_MOESM2_ESM.pdf]

Description of Additional Supplementary Files

File Name: Supplementary Movie 1
Description: Real-time operation of the epi-GLIM system. The user shifts through the intensity frames, activates the real-time reconstruction, and applies hybrid denoising to obtain an image that has substantially higher contrast than regular reflection DIC. For demonstration purposes, we used thinly sliced resected pathology tissue (40x/0.75NA objective).

File Name: Supplementary Movie 2
Description: Epi-GLIM enables wafer-scale QPI. Large field of view assembled from (60 x 83 tiles of 830 x 830 µm2) at 10x/0.3NA using alignment software developed in-house.

File Name: Supplementary Movie 3
Description: Larval zebrafish showing blood circulation. Imaging took place approximately seven days post fertilization (10x/0.3NA objective).
